# ETHNICITY, RACE, HEALTH, AND SOCIAL CARE: STUDYING INEQUALITIES IN AN INJUSTICE-OBLIVIOUS WAY

**DOI:** 10.1093/geroni/igz038.712

**Published:** 2019-11-08

**Authors:** Sandra Torres

**Affiliations:** Uppsala Universitet, UPPSALA, Sweden

## Abstract

Scholarship on ethnicity and old age is at a crossroad now that increased diversity is a given in older populations. The same holds true for the study of inequalities in old age as it relates to ethnicity and race. This presentation relies on a scoping review of scholarship published between 1998 - 2017 (n=336) that brings attention to the ways in which ethnicity/ race – as grounds for stratification and disadvantage - are made sense of in this scholarship. The presentation will describe the topics that the review divulged when it comes to the study of health and social care (i.e. access and usage; attitudes, preferences and experiences; assessment of programs suitability and self-care practices). In doing so, this presentation will argue that if we are to address the inequalities that older ethnic minorities face we need not only a diversity-astute research agenda but also an injustice-aware one.

